# Supplementary material for: Pyruvate Dehydrogenase Kinase 1 inhibition mediated oxidative phosphorylation enhancement in cartilage promotes osteoarthritis progression
Source: BMC Musculoskelet Disord. 2023 Jul 20;24:597. doi: 10.1186/s12891-023-06585-6 (PMC10357736; doi:10.1186/s12891-023-06585-6)
Supplement: Supplementary file 1 — Additional file 1. [file 12891_2023_6585_MOESM1_ESM.zip › MF.Gsea.1653627316242/GOMF_E_BOX_BINDING.html]

Details for gene set GOMF\_E\_BOX\_BINDING[GSEA]

|  || Dataset | 55457\_collapsed\_to\_symbols.cls.cls #OA\_versus\_normal.cls.cls #OA\_versus\_normal\_repos |
| Phenotype | cls.cls#OA\_versus\_normal\_repos |
| Upregulated in class | normal |
| GeneSet | GOMF\_E\_BOX\_BINDING |
| Enrichment Score (ES) | -0.42906615 |
| Normalized Enrichment Score (NES) | -1.5857623 |
| Nominal p-value | 0.011824325 |
| FDR q-value | 0.15343508 |
| FWER p-Value | 0.998 |
Table: GSEA Results Summary

  

Fig 1: Enrichment plot: GOMF\_E\_BOX\_BINDING      
 Profile of the Running ES Score & Positions of GeneSet Members on the Rank Ordered List

  

| SYMBOL | TITLE | RANK IN GENE LIST | RANK METRIC SCORE | RUNNING ES | CORE ENRICHMENT || 1 | BHLHE41 | basic helix-loop-helix family member e41 [Source:HGNC Symbol;Acc:HGNC:16617] | 417 | 0.700 | 0.0165 | No |
| 2 | ARNTL2 | aryl hydrocarbon receptor nuclear translocator like 2 [Source:HGNC Symbol;Acc:HGNC:18984] | 937 | 0.531 | 0.0133 | No |
| 3 | NEUROD1 | neuronal differentiation 1 [Source:HGNC Symbol;Acc:HGNC:7762] | 1008 | 0.515 | 0.0439 | No |
| 4 | CLOCK | clock circadian regulator [Source:HGNC Symbol;Acc:HGNC:2082] | 3231 | 0.227 | -0.1130 | No |
| 5 | MAX | MYC associated factor X [Source:HGNC Symbol;Acc:HGNC:6913] | 3804 | 0.176 | -0.1452 | No |
| 6 | MYF6 | myogenic factor 6 [Source:HGNC Symbol;Acc:HGNC:7566] | 4057 | 0.150 | -0.1543 | No |
| 7 | TCF21 | transcription factor 21 [Source:HGNC Symbol;Acc:HGNC:11632] | 4321 | 0.127 | -0.1659 | No |
| 8 | MITF | melanocyte inducing transcription factor [Source:HGNC Symbol;Acc:HGNC:7105] | 4759 | 0.090 | -0.1936 | No |
| 9 | ASCL2 | achaete-scute family bHLH transcription factor 2 [Source:HGNC Symbol;Acc:HGNC:739] | 5194 | 0.057 | -0.2233 | No |
| 10 | TAL1 | "TAL bHLH transcription factor 1, erythroid differentiation factor [Source:HGNC Symbol;Acc:HGNC:11556]" | 5406 | 0.040 | -0.2370 | No |
| 11 | PPARG | peroxisome proliferator activated receptor gamma [Source:HGNC Symbol;Acc:HGNC:9236] | 6006 | -0.005 | -0.2832 | No |
| 12 | TCF12 | transcription factor 12 [Source:HGNC Symbol;Acc:HGNC:11623] | 6398 | -0.035 | -0.3112 | No |
| 13 | TFAP4 | transcription factor AP-4 [Source:HGNC Symbol;Acc:HGNC:11745] | 6823 | -0.065 | -0.3396 | No |
| 14 | NR1D1 | nuclear receptor subfamily 1 group D member 1 [Source:HGNC Symbol;Acc:HGNC:7962] | 6974 | -0.078 | -0.3458 | No |
| 15 | ASCL1 | achaete-scute family bHLH transcription factor 1 [Source:HGNC Symbol;Acc:HGNC:738] | 7375 | -0.108 | -0.3694 | No |
| 16 | TCF3 | transcription factor 3 [Source:HGNC Symbol;Acc:HGNC:11633] | 7521 | -0.120 | -0.3723 | No |
| 17 | MYOD1 | myogenic differentiation 1 [Source:HGNC Symbol;Acc:HGNC:7611] | 7839 | -0.143 | -0.3869 | No |
| 18 | PRMT5 | protein arginine methyltransferase 5 [Source:HGNC Symbol;Acc:HGNC:10894] | 7904 | -0.148 | -0.3815 | No |
| 19 | HDAC1 | histone deacetylase 1 [Source:HGNC Symbol;Acc:HGNC:4852] | 7911 | -0.148 | -0.3716 | No |
| 20 | ZEB1 | zinc finger E-box binding homeobox 1 [Source:HGNC Symbol;Acc:HGNC:11642] | 8147 | -0.166 | -0.3783 | No |
| 21 | HAND2 | heart and neural crest derivatives expressed 2 [Source:HGNC Symbol;Acc:HGNC:4808] | 8211 | -0.172 | -0.3711 | No |
| 22 | NEUROD2 | neuronal differentiation 2 [Source:HGNC Symbol;Acc:HGNC:7763] | 8707 | -0.210 | -0.3949 | No |
| 23 | TCF15 | transcription factor 15 [Source:HGNC Symbol;Acc:HGNC:11627] | 8925 | -0.226 | -0.3960 | No |
| 24 | AHR | aryl hydrocarbon receptor [Source:HGNC Symbol;Acc:HGNC:348] | 9263 | -0.253 | -0.4045 | Yes |
| 25 | NEUROG2 | neurogenin 2 [Source:HGNC Symbol;Acc:HGNC:13805] | 9393 | -0.263 | -0.3962 | Yes |
| 26 | MYF5 | myogenic factor 5 [Source:HGNC Symbol;Acc:HGNC:7565] | 9572 | -0.280 | -0.3904 | Yes |
| 27 | SNAI2 | snail family transcriptional repressor 2 [Source:HGNC Symbol;Acc:HGNC:11094] | 9697 | -0.291 | -0.3797 | Yes |
| 28 | NEUROG1 | neurogenin 1 [Source:HGNC Symbol;Acc:HGNC:7764] | 9856 | -0.307 | -0.3706 | Yes |
| 29 | TCF4 | transcription factor 4 [Source:HGNC Symbol;Acc:HGNC:11634] | 9918 | -0.313 | -0.3534 | Yes |
| 30 | SREBF2 | sterol regulatory element binding transcription factor 2 [Source:HGNC Symbol;Acc:HGNC:11290] | 10070 | -0.328 | -0.3422 | Yes |
| 31 | MYBBP1A | MYB binding protein 1a [Source:HGNC Symbol;Acc:HGNC:7546] | 11188 | -0.456 | -0.3972 | Yes |
| 32 | CRY1 | cryptochrome circadian regulator 1 [Source:HGNC Symbol;Acc:HGNC:2384] | 11247 | -0.463 | -0.3693 | Yes |
| 33 | TWIST1 | twist family bHLH transcription factor 1 [Source:HGNC Symbol;Acc:HGNC:12428] | 11696 | -0.531 | -0.3670 | Yes |
| 34 | HIF1A | hypoxia inducible factor 1 subunit alpha [Source:HGNC Symbol;Acc:HGNC:4910] | 11833 | -0.556 | -0.3387 | Yes |
| 35 | ARNTL | aryl hydrocarbon receptor nuclear translocator like [Source:HGNC Symbol;Acc:HGNC:701] | 12120 | -0.625 | -0.3172 | Yes |
| 36 | GATA3 | GATA binding protein 3 [Source:HGNC Symbol;Acc:HGNC:4172] | 12276 | -0.670 | -0.2824 | Yes |
| 37 | MYOG | myogenin [Source:HGNC Symbol;Acc:HGNC:7612] | 12313 | -0.681 | -0.2376 | Yes |
| 38 | SNAI1 | snail family transcriptional repressor 1 [Source:HGNC Symbol;Acc:HGNC:11128] | 12365 | -0.696 | -0.1929 | Yes |
| 39 | PER1 | period circadian regulator 1 [Source:HGNC Symbol;Acc:HGNC:8845] | 12496 | -0.746 | -0.1507 | Yes |
| 40 | BHLHE40 | basic helix-loop-helix family member e40 [Source:HGNC Symbol;Acc:HGNC:1046] | 12573 | -0.782 | -0.1019 | Yes |
| 41 | HES1 | hes family bHLH transcription factor 1 [Source:HGNC Symbol;Acc:HGNC:5192] | 12676 | -0.849 | -0.0505 | Yes |
| 42 | MYC | "MYC proto-oncogene, bHLH transcription factor [Source:HGNC Symbol;Acc:HGNC:7553]" | 12801 | -0.970 | 0.0077 | Yes |
Table: GSEA details [plain text format]

  

Fig 2: GOMF\_E\_BOX\_BINDING      
 Blue-Pink O' Gram in the Space of the Analyzed GeneSet

  

Fig 3: GOMF\_E\_BOX\_BINDING: Random ES distribution      
 Gene set null distribution of ES for **GOMF\_E\_BOX\_BINDING**
